# Evidence-based Tool for Triggering School Closures during Influenza Outbreaks, Japan

**DOI:** 10.3201/eid1511.090798

**Published:** 2009-11

**Authors:** Asami Sasaki, Anne Gatewood Hoen, Al Ozonoff, Hiroshi Suzuki, Naohito Tanabe, Nao Seki, Reiko Saito, John S. Brownstein

**Affiliations:** Harvard School of Public Health, Boston, Massachusetts, USA (A. Sasaki); University of Niigata Prefecture, Niigata, Japan (A. Sasaki); Children’s Hospital, Boston (A. Gatewood Hoen, J.S. Brownstein); Harvard Medical School, Boston (A. Gatewood Hoen, J.S. Brownstein); Boston University School of Public Health, Boston (A. Ozonoff); Niigata University Graduate School of Medical and Dental Sciences, Niigata (H. Suzuki, N. Tanabe, N. Seki, R. Saito)

**Keywords:** Outbreaks, influenza, population surveillance, software design, public health, prevention and control, expedited, schools, Japan, dispatch

## Abstract

Guidelines available to school administrators to support school closure decisions during influenza outbreaks are usually not evidence-based. Using empirical data on absentee rates of elementary school students in Japan, we developed a simple and practical algorithm for determining the optimal timing of school closures for control of influenza outbreaks.

Influenza pandemic preparedness and seasonal influenza control programs have focused on vaccine development and antiviral drugs, which are only partially effective and not always available to all persons at risk (*1**–**3*). Nonpharmaceutical interventions, such as social distancing, represent additional key tools for mitigating the impact of outbreaks. Because children are a major factor in the transmission of influenza within communities and among households, school closure may be a valuable social distancing method (*4**,**5*).

Japan has a unique system of monitoring school absenteeism and of instituting school closures during influenza outbreaks. Individual classes, specific grade levels, or the entire school may be closed; final decision-making authority is given to school principals. However, as in the United States and other countries, there are no regulations to support these decisions (*6*). Our study suggests a simple system to help determine when schools should be closed; daily influenza-related absentee thresholds are measured to predict outbreaks.

## The Study

We used data on absenteeism caused by influenza from the 54 elementary schools in Joetsu City, Niigata Prefecture, Japan during the 4 influenza seasons during 2005–2008. Data was obtained between the second week of January to the third week of March for each influenza season. Average school size was 221 students. Current public health policy prevents influenza-infected children from attending school until 2 days after fever has disappeared. An illness requires 2 physician visits: 1 for the initial diagnosis and 1 to obtain written permission from the treating physician to return to school. Diagnoses are usually made by using a rapid antigen test and patients are treated with the antiviral drugs, oseltamivir or zanamivir.

**Figure 1:**
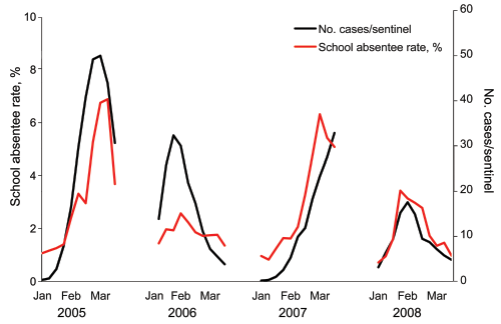
Four-year surveillance of influenza-related absentee rates in 54 elementary schools in Joetsu City and national surveillance of influenza-like illness (ILI) reported by sentinel physicians in Japan. Data were collected from the second week of January (after the winter holiday) to the third week of March (before the spring holiday). The average of the daily absentee rates for 54 elementary schools during 4 influenza seasons (2005–2008) were 3.29%, 1.77%, 2.97%, and 1.92%, respectively.

Based on elementary school daily influenza-related absentee surveillance, the most intense influenza seasons were 2005 and 2007 (Figure 1). The number of schools reporting outbreaks during the 4 influenza seasons was 34 (63%, 2005), 13 (24%, 2006), 35 (65%, 2007) and 18 (33%, 2008), respectively. Rates of absenteeism caused by confirmed influenza infection in the 54 elementary schools in Joetsu City were well correlated with national reports of influenza-like illness by 5,000 sentinel physicians, who reported 322, 205, 226, and 142 cumulative cases of infection per sentinel in each season (Technical Appendix).

We evaluated the optimal influenza-related absentee rate for predicting outbreaks of influenza. For this study, we defined an influenza outbreak in a school as a daily influenza-related absentee rate of >10%, on the basis of the 95th percentile of daily absentee rates (10.7%) in 54 elementary schools during 4 influenza seasons (Technical Appendix).

Next, we considered 9 different daily influenza-related absentee threshold levels for initiating early school closures: 1%, 2%, 3%, … , 9%. In addition, for each threshold level, we considered 3 scenarios: 1) a single-day scenario, in which daily influenza-related absentee rates are observed for the first time above a given threshold for 1 day; 2) a double-day scenario, in which rates reached a given threshold for the first time for 2 consecutive days; the rate for the second day was the same or higher than for the first day; and 3) a triple-day scenario, in which rates reached a given threshold for the first time for 3 consecutive days; rates for the second and third days were the same or higher than the rate for the first day. The double-day and triple-day scenarios did not include weekends. To evaluate the performance of prediction for each threshold, we determined the school’s outbreak status in the 7-day period starting on the first day of each scenario (Technical Appendix) JMP7.0.1 (SAS Institute, Inc., Cary, NC, USA) was used for statistical analysis.

**Figure 2:**
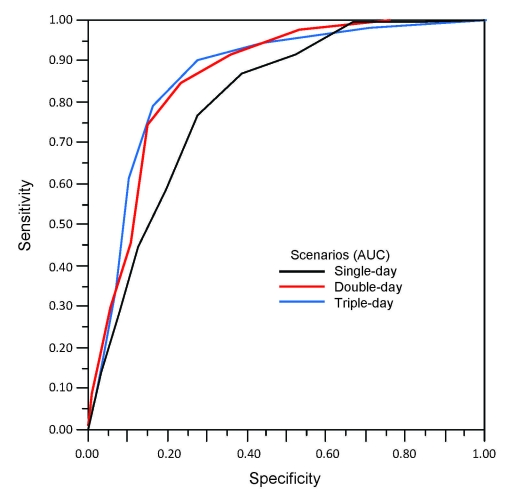
The receiver operating characteristic (ROC) curve for detection of influenza outbreak by 1%–9% thresholds under single-day, double-day, triple-day scenarios. ROC space is defined on the x axis as 1 – specificity and on the y axis as sensitivity. The area under the curve (AUC) is an indicator of the quality of a model; larger AUC values corresponded to better performance. Optimal thresholds for the 3 scenarios are *single-day, 5%; †double-day, 4%; and ‡triple-day, 3%.

We calculated the sensitivity and specificity of each scenario at all 9 threshold levels, and presented these data as a plot in Figure 2. The area under the curve for the single-, double-, and triple-day scenarios was 0.80 (95% confidence interval [CI] 0.77–0.83), 0.85 (95% CI 0.82–0.89) and 0.87 (95% CI 0.83–0.91), respectively.

We used the Youden index for calculating optimal thresholds (*7*). The Youden index = (sensitivity) + (specificity) – 1. A perfect test result would have a Youden index of 1. For the single-day scenario, the optimal threshold was 5%, with a sensitivity of 0.77 and specificity of 0.73. For the double-day scenario, the optimal threshold was 4%, with a sensitivity of 0.84 and specificity of 0.77. For the triple-day scenario, the optimal threshold was 3%, with a sensitivity of 0.90 and specificity of 0.72.

## Conclusions

We have demonstrated the predictive value of a simple and practical detection method for triggering school closures early after influenza outbreaks. Our analysis suggests that a single-day at a threshold influenza-related absentee rate of 5%, double-days >4%, or triple-days >3% are optimal levels for alerting school administrators to consider school closure. The double- and triple-day scenarios performed similarly, and gave better results than the single-day. Thus, the double-day scenario might be the preferred early warning trigger.

Our study had the advantage of reliable empirical data on influenza-related absenteeism in schools. Data were based on physician and laboratory diagnosis and a strong absentee surveillance program. However, there are limitations to our approach. We did not have available vaccination or medication histories of patients. Also, our results are based on data from only 1 city’s school district; validation in a broader area will be required. Although separate analyses may be required for other geographic regions, we present a simple approach that can be easily reapplied.

Influenza outbreak detection from surveillance data typically relies on relatively complex time series analysis or smoothing (*8**,**9*). The noisiness of school surveillance data makes detection of outbreaks difficult (*10*). However, complex statistical analyses are not practical to use in the context of daily decision-making in schools. Despite the limitations of our study, we have presented a method that provides a basis for empirical data-supported decision-making by school administrators that is intuitive and practical.

School closure could be an effective method of social distancing, although evidence supporting its effectiveness is incomplete. Some studies suggest that though child-to-child transmission might decrease, transmission might increase in other age groups (*11**,**12*). During school closures, children may need to forgo participation in external activities that could increase contact rates. Additionally, working parents staying home to care for their children (*13*) could result in a decrease in household income, causing loss of productivity and economic losses (*14*). Decision-makers will need to consider these factors when considering school closures.

During the early days of the outbreak of influenza A pandemic (H1N1) 2009 virus, the US Centers for Disease Control and Prevention (Atlanta, GA, USA) released 2 different recommendations for school dismissal after the appearance of the first suspected case: dismiss for 7 days (as of April 26) and then for 14 days (as of May 1). Later, to reflect new knowledge about the extent of community spread and disease severity, the recommendation was revised to advise against school closure unless absentee rates interfered with school function (*15*). The pandemic (H1N1) 2009 influenza outbreak highlights the need for a flexible national policy that can be quickly adapted to reflect current situations. The evidence-based strategy for predicting outbreaks based on influenza-related absentee rates that we present here provides local administrators, who may need to consider school closure, with a simple and practical tool to aid in their decisions.

## Supplementary Material

Technical Appendix Evidence-based Tool for Triggering School Closures during Influenza Outbreaks, Japan
