# Supplementary material for: Evidence-based Tool for Triggering School Closures during Influenza Outbreaks, Japan
Source: Emerg Infect Dis. 2009 Nov;15(11):1841–3. doi: 10.3201/eid1511.090798 (PMC2857248; doi:10.3201/eid1511.090798)
Supplement: Technical Appendix — Evidence-based Tool for Triggering School Closures during Influenza Outbreaks, Japan [file 09-0798_Techapp-s1.pdf]

Evidence-based Tool for Triggering School Closures during Influenza Outbreaks, Japan

Technical Appendix

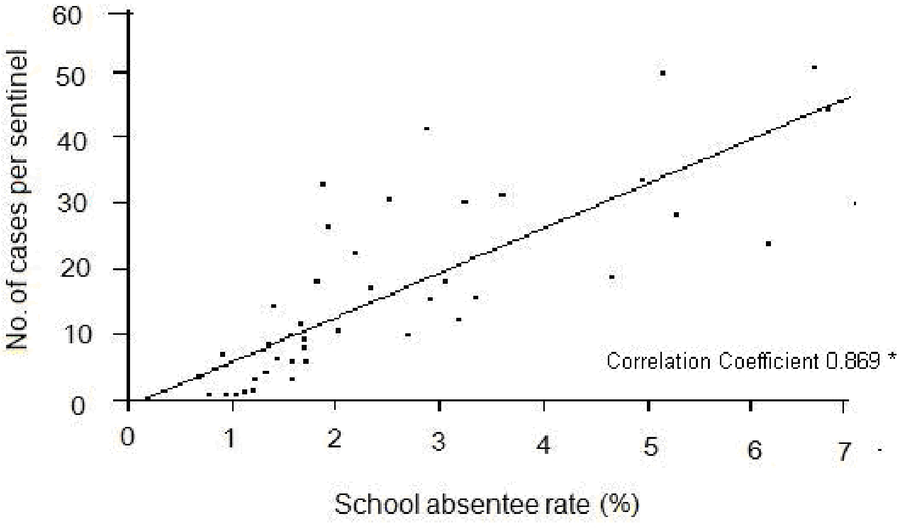

Technical Appendix Figure 1. Plot of weekly absentee rates of influenza cases from 54 elementary schools vs. weekly national influenza-like-illness (ILI) cases reported by Sentinel physicians, 2005–2008. *p<0.01.

Relation between national surviellance with +1 week lag and school surveillance

Correlations

			sentinel	schoolaverag erate
Spearman's rho	sentinel	Correlation Coefficient	1.000	.753**
		Sig. (2-tailed)	.	.000
		N	40	40
	schoolaveragerate	Correlation Coefficient	.753**	1.000
		Sig. (2-tailed)	.000	.
		N	40	40

**. Correlation is significant at the 0.01 level (2-tailed).

Relation between school surveillance with+ 1 week lag and national surviellance

			sentinel	schoolaverag erate
Spearman's rho	sentinel	Correlation Coefficient	1.000	.806**
		Sig. (2-tailed)	.	.000
		N	39	39
	schoolaveragerate	Correlation Coefficient	.806**	1.000
		Sig. (2-tailed)	.000	.
		N	39	39

**. Correlation is significant at the 0.01 level (2-tailed).

Relation between national surviellance with+2 week lag and school surveillance

Correlations

			sentinel	schoolaverag erate
Spearman's rho	sentinel	Correlation Coefficient	1.000	.587**
		Sig. (2-tailed)	.	.000
		N	35	35
	schoolaveragerate	Correlation Coefficient	.587**	1.000
		Sig. (2-tailed)	.000	.
		N	35	35

**. Correlation is significant at the 0.01 level (2-tailed).

Relation between school surveillance with+2 week lag and national surviellance

Correlations

			sentinel	schoolaverag erate
Spearman's rho	sentinel	Correlation Coefficient	1.000	.452**
		Sig. (2-tailed)	.	.003
		N	41	41
	schoolaveragerate	Correlation Coefficient	.452**	1.000
		Sig. (2-tailed)	.003	.
		N	41	43

**. Correlation is significant at the 0.01 level (2-tailed).

Technical Appendix Figure 2. Correlation between lagged weekly rates of absenteeism due to confirmed influenza cases from 54 elementary schools and weekly national influenza-like-illness cases reported by Sentinel physicians, 2005–2008. None of the lagged comparisons resulted in an improved correlation over the unlagged relationship shown in Technical Appendix Figure 1.

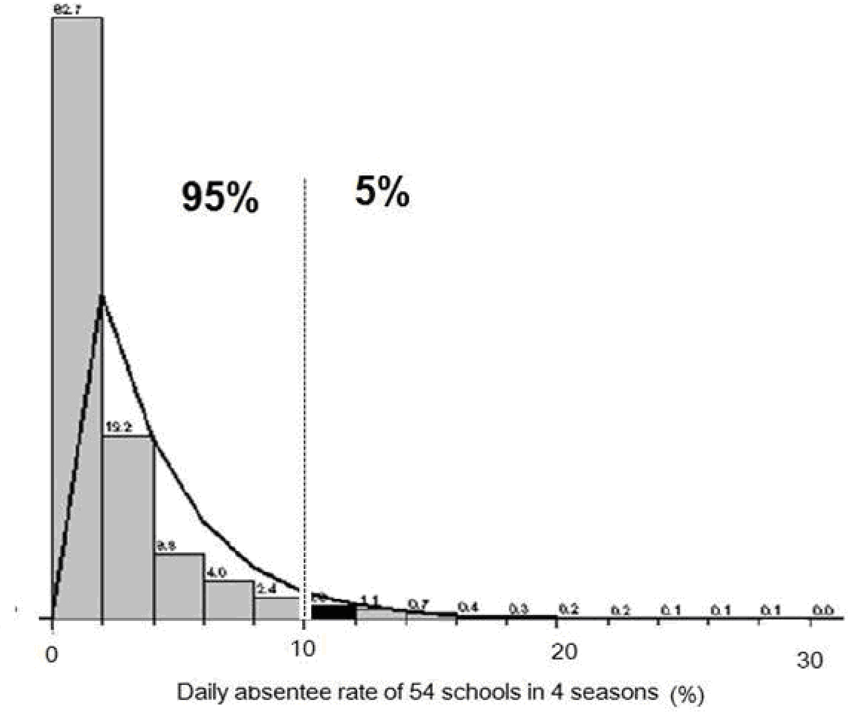

Technical Appendix Figure 3. Histogram of daily rate of absenteeism related to confirmed influenza cases in 54 elementary schools. We defined an influenza outbreak in a school as a daily influenza-related absentee rate of >10%, on the basis of the 95th percentile of daily absentee rates (10.7%) for 4 influenza seasons.

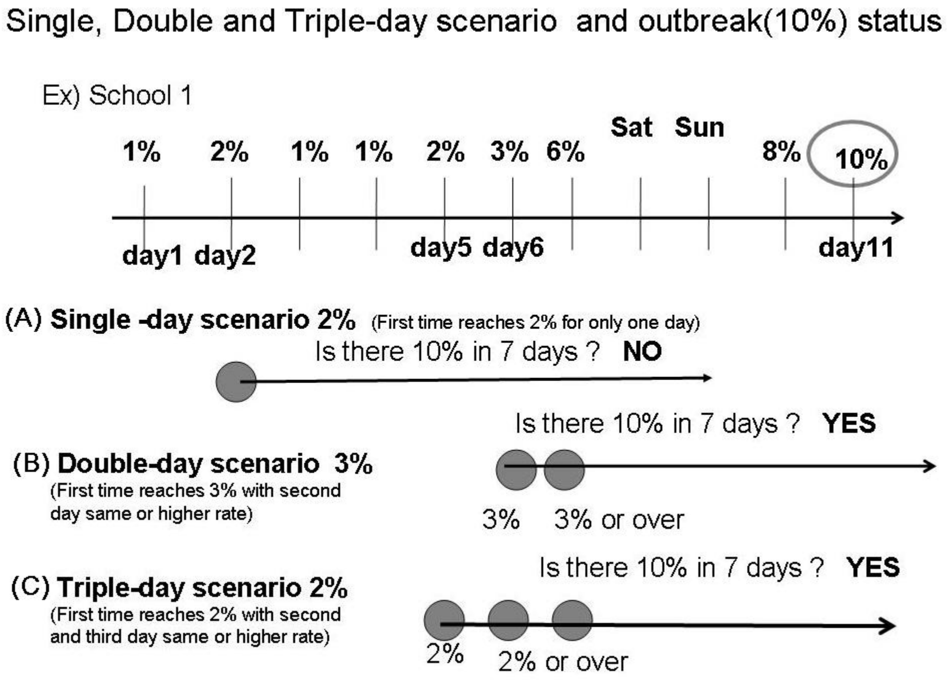

Technical Appendix Figure 4. Schematic illustration of our method for evaluating and optimizing our algorithm. For each of the 54 elementary schools, and for each influenza season of the study, we considered 3 scenarios: a single-day scenario, in which daily influenza-related absentee rates are observed for the first time above a given threshold for 1 day; a double-day scenario, in which rates reached a given threshold for the first time for 2 consecutive days, with the second day at the same rate or higher than the first; and a triple-day scenario, in which rates reached a given threshold for the first time for 3 consecutive days, with the second and third days at the same rate or higher than the first. Each scenario was evaluated at 9 different absentee threshold points: 1%, 2% … 9%. The example illustrated above shows how we evaluated the algorithm at 1 school during 1 influenza season under 3 arbitrarily chosen scenario-threshold combinations. A) For the single-day scenario evaluated at the 2% threshold, we calculated the date that absenteeism due to confirmed influenza reached at least 2% and noted whether the outbreak threshold of 10% was reached in the following 7 days. B) For the double-day scenario evaluated at the 3% threshold level, we calculated the date that absenteeism due to confirmed influenza reached at least 3% and was sustained at $\geq$3% for at least 2 consecutive days (excluding weekends), and then noted whether the outbreak threshold of 10% was reached within the 7 days after the first day. C) For the triple-day scenario evaluated at the 2% threshold level, we calculated the date that absenteeism due to confirmed influenza reached at least 2% and was sustained at $\geq$2% for at least 3 consecutive days (excluding weekends), and then noted whether the outbreak threshold of 10% was reached in the 7 days after the first day.
